# Osteopathic Legislation for District of Columbia

**Published:** 1907-03

**Authors:** 


### OSTEOPATHIC LEGISLATION FOR DISTRICT OF COLUMBIA.

Senate Bill, No. 5221, introduced by Senator Foraker, Ohio, has passed the U. S. Senate and is now before a committee of the House of Representatives of which the Hon. A. J. Barchfield (Penn.) is chairman.

The objections urged against this bill by the Medical Society of the District of Columbia and ratified by the National Legislative Council of the American Medical Association are as follows:—

"First.—The bill, if enacted, will lower all existing medical educational standards and thus defeat the very object for which medical practice laws are provided, viz., to guard the people against the effects of ignorance and incompetency in the practice of medicine.

"Second.—The bill seeks to legalize and regulate the practice of osteopathy without defining what constitutes the practice of osteopathy, and until this is done Congress can not undertake to legislate intelligently.

"Third.—The bill, by establishing special provisions for a special group of physicians as distinguished from other groups of physicians, contemplates class legislation."

Every physician is requested to forward at once his protest to the Hon. A. J. Barchfield, House of Representatives, Washington, D. C., and also secure on such protests the signatures of prominent citizens and politicians. What is done must be done at once. If you make no effort along this line, let your voice never be heard croaking about the lack of influence of the profession. Blank petitions to be signed in protest of the passage of this bill will be furnished by the writer on request.

The osteopaths are earnestly soliciting signatures to their petitions, claiming that all they ask is to have the same chance as others. They are, however, seeking very much more. Physicians now are required to have a good preliminary education before spending four years in a medical college in order to make them eligible to an examination for licensure to practice medicine. The osteopaths ask that those now practicing osteopathy be licensed. This is class legislation of the worst kind. Much more reasonable would it be for qualified dentists or veterinary surgeons to ask to be legalized as practitioners of medicine.

The Pittsburg Dispatch for July 29, 1906, contained the following advertisement:—

"PERSONAL.—All persons practicing osteopathy in Pennsylvania (with or without diploma), who are desirous of having a liberal law enacted, write at once to The American Osteopathic Union, 600 and 601 Lewis Block, Pittsburg."

The words "liberal law" and "with or without a diploma" explain the activity of these people in circulating petitions, and their readiness to raise a large fund for legislative purposes. Before this article will be read a bill will have been introduced into the Pennsylvania Legislature to legalize the practice of osteopathy. Success in the District of Columbia would give them a precedent sufficient to carry through their bill at Harrisburg.—Pennsylvania Medical Journal, January, 1907.

---

The "bends," caisson disease, or diver's palsy has been known so long and its cause and prevention have been so thoroughly explained, that it is surprising there should be any cases, at least any fatalities. The extensive operations under the New York rivers in constructing tunnels and bridge piers, are producing many fatal cases and we must expect more in the future if greater precautions are not taken. The cause of the disease is purely mechanical. Gases dissolve in liquids in proportion to the pressure, as in a soda water bottle. If the pressure is very slowly relieved the gases escape by an invisible evaporation, but if the cork is suddenly drawn, they flash out in bubbles. Men working in high-air pressure behave in the same way. The blood absorbs a great deal of air, and it is entirely harmless; and if the pressure is very slowly relieved, the extra gases evaporate invisibly and so quietly that no one knows he was "charged."

The cause of "bends," then, is a too rapid relief of the pressure so that bubbles of air form in every part of the body where liquids are found—heart, vessels, serous cavities, and especially around the brain and nerves. Death is instantaneous if the blood current is thus plugged by a froth or if the cardiac or respiratory gangions are choked. In less severe cases, only small areas are deprived of blood-supply and the circulation is often restored as the gases disappear, by a reabsorption under normal pressure. Nerves are sometimes so injured that they never recover and permanent paralysis result. The treatment is the immediate restoration of the patient to the original high pressure, though even this will sometimes fail to redissolve the bubbles. Prevention is the slow relief of the pressure. Workmen, on coming out of the caissons, first enter an intermediate room or lock where the pressure is so slowly reduced that the gases evaporate harmlessly from the fluid tissues.

The loss of time in preventing "bends" is resented by both workmen and the construction companies. It is said that fifteen minutes in the lock for every atmosphere of pressure is sufficient, but the fatalities show that it is not or that there has been a violation of the rules. Something is wrong--that much is certain—but whether there has been criminal carelessness only an official investigation will tell. Certainly it is a traumatism which physicians never should see; but with our strenuosity, and contempt of life, it takes its place with that very long list of preventable accidents for which America is famous. Perhaps some amelioration of the condition might result from stirring up public sentiment on the subject.—Amer. Med., Nov., 1906.

